# Ionizable lipid nanoparticles deliver mRNA to pancreatic β cells via macrophage-mediated gene transfer

**DOI:** 10.1126/sciadv.ade1444

**Published:** 2023-01-27

**Authors:** Jilian R. Melamed, Saigopalakrishna S. Yerneni, Mariah L. Arral, Samuel T. LoPresti, Namit Chaudhary, Anuradha Sehrawat, Hiromi Muramatsu, Mohamad-Gabriel Alameh, Norbert Pardi, Drew Weissman, George K. Gittes, Kathryn A. Whitehead

**Affiliations:** ^1^ Department of Chemical Engineering, Carnegie Mellon University, Pittsburgh, PA 15213, USA.; ^2^ Department of Medicine, University of Pennsylvania, Philadelphia, PA 19104, USA.; ^3^ Department of Pediatric Surgery, Department of Surgery, Children’s Hospital of Pittsburgh, University of Pittsburgh School of Medicine, Pittsburgh, PA 15224, USA.; ^4^ Department of Microbiology, Perelman School of Medicine, University of Pennsylvania, Philadelphia, PA 19104, USA.; ^5^ Department of Biomedical Engineering, Carnegie Mellon University, Pittsburgh, PA 15213, USA.

## Abstract

Systemic messenger RNA (mRNA) delivery to organs outside the liver, spleen, and lungs remains challenging. To overcome this issue, we hypothesized that altering nanoparticle chemistry and administration routes may enable mRNA-induced protein expression outside of the reticuloendothelial system. Here, we describe a strategy for delivering mRNA potently and specifically to the pancreas using lipid nanoparticles. Our results show that delivering lipid nanoparticles containing cationic helper lipids by intraperitoneal administration produces robust and specific protein expression in the pancreas. Most resultant protein expression occurred within insulin-producing β cells. Last, we found that pancreatic mRNA delivery was dependent on horizontal gene transfer by peritoneal macrophage exosome secretion, an underappreciated mechanism that influences the delivery of mRNA lipid nanoparticles. We anticipate that this strategy will enable gene therapies for intractable pancreatic diseases such as diabetes and cancer.

## Teaser

Lipid nanoparticles deliver mRNA to insulin-producing cells in the pancreas.

## INTRODUCTION

mRNA therapeutics have virtually limitless clinical potential for vaccination (*1*–*5*), protein replacement (*6*, *7*), gene editing (*8*–*10*), immunotherapies (*11*–*13*), and tissue regeneration (*14*, *15*). This clinical utility, particularly for mRNA vaccines, is evidenced by their suppression of the ongoing SARS-CoV-2 pandemic (*16*, *17*). Given this success, we anticipate a surge in the clinical translation of mRNA therapies for other applications. However, to fully unlock the potential of mRNA drugs, the field must first develop delivery systems that access diseased cells and tissues.

Delivery systems are needed because “naked” mRNA has unfavorable pharmacokinetic properties and is rapidly degraded and cleared before it reaches the cytoplasm of target cells, where translation into functional protein occurs (*18*). Efficient delivery systems must protect the mRNA from nuclease degradation in the body, induce uptake by target cells, and release the mRNA from endosomes into the cytoplasm. Although lipid nanoparticles (LNPs) are proven RNA delivery systems in humans (*4*, *5*, *10*, *19*), most of them are limited in their ability to deliver mRNA to cellular targets outside immune cells and hepatocytes. Vaccines effectively deliver mRNA to immune cells by intramuscular injection, while protein replacement and immunotherapies typically require intravenous administration. For example, the Food and Drug Administration–approved small interfering RNA (siRNA) LNP drug, patisiran, treats hereditary transthyretin amyloidosis following intravenous delivery to hepatocytes (*19*). In addition, a recent clinical trial conducted by Intellia Therapeutics and Regeneron yielded positive data using RNA-LNP–mediated gene editing to cure amyloidosis (*10*). These successes highlight the potential for RNA-LNPs to treat disease. However, little progress has been made toward achieving systemic RNA delivery to organs outside the liver and spleen.

Several aspects of mRNA-LNP design and delivery can be tuned in pursuit of transfecting difficult cellular targets, including the lipid components (*20*–*22*), mRNA sequence (*23*–*29*), route of administration (*30*), and incorporation of active targeting agents (*12*, *31*, *32*). LNPs typically contain four lipid components: an ionizable lipid, an amphipathic phospholipid (i.e., helper lipid), cholesterol, and poly(ethylene glycol) (PEG) lipids (*18*, *33*). The ionizable lipid has been the primary focus of LNP development, as it plays a key role in facilitating endosomal escape by protonating as the local pH drops within endosomes. However, screens of large libraries have identified few materials that deliver mRNA outside of the liver and spleen (*8*, *20*–*22*, *34*). Alternative administration routes can circumvent this issue, as has been demonstrated by our laboratory (*35*) and others (*30*) for mRNA delivery to organs including the heart (*36*), brain (*37*), and lungs (*38*, *39*). Extrahepatic mRNA delivery can also be achieved by incorporating charged amphipathic phospholipids, which shifts protein expression away from the liver to the spleen or lungs (*40*–*42*).

Despite these advances, the delivery of mRNA-LNPs to cells in the pancreas remains challenging. Such a therapeutic could provide lifesaving treatments for incurable pancreatic diseases such as cancer and diabetes. The pancreas performs both endocrine and exocrine functions; cells in the islets of Langerhans are responsible for maintaining glucose homeostasis, while acinar cells secrete digestive enzymes into the duodenum. The clinical potential of pancreatic mRNA delivery has already been demonstrated using viral delivery systems. For example, Gittes and colleagues showed that viral gene delivery to the pancreas regenerated insulin-producing β cells as a therapy for autoimmune diabetes (*43*). While viral vectors are efficient cellular transducers, they risk integrating into the host genome and are highly immunogenic, which limits their capacity for repeat dosing (*44*). Furthermore, their therapeutic use in the pancreas requires injection through the pancreatic duct using endoscopic retrograde cholangiopancreatography, which is invasive and risks inducing pancreatitis (*45*).

As an alternative to viral gene therapy, we were motivated to develop mRNA-LNPs to enable non-viral gene delivery to the pancreas. We further wanted to reduce the invasiveness of the required infusion procedure. To accomplish this, we turned to intraperitoneal injection, which is an effective strategy for selectively delivering drugs to disease sites in the peritoneal cavity such as ovarian and pancreatic tumors (*46*). In contrast to intravenous delivery, intraperitoneal administration may reduce systemic toxicity, provide greater bioavailability, and prolong contact with peritoneal organ targets due to the high retention of nanoparticles within the peritoneal cavity (*47*, *48*).

Here, we describe an LNP formulation that potently and selectively delivers mRNA to the pancreas. We show that intraperitoneal delivery of mRNA-LNPs induces robust protein expression in the pancreas for structurally distinct ionizable lipids. This strategy induced protein expression primarily in β cells, which are insulin-producing cells located in pancreatic islets. Furthermore, we show that mRNA delivery is facilitated by peritoneal macrophage extracellular vesicle (EV) transfer and that efficacy is not linked to systemic toxicity. Together, these data suggest that mRNA-LNPs are a viable, non-viral means of inducing protein expression in difficult-to-transfect pancreatic cells.

## RESULTS

### Intraperitoneal administration facilitates mRNA delivery to the pancreas

**Fig. 1.**
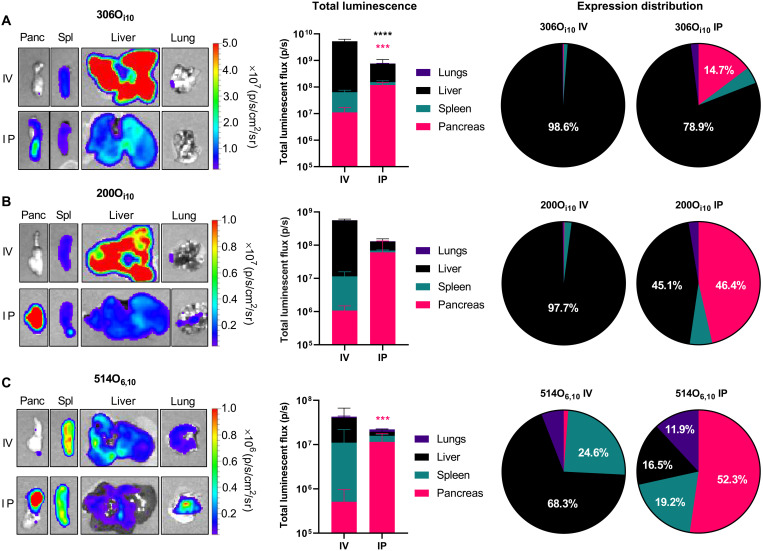
Intraperitoneal administration improves pancreatic mRNA delivery relative to intravenous injection. LNPs containing mLuc were formulated using each of three ionizable lipidoids—(**A**) 306O_i10_, (**B**) 200O_i10_, or (**C**) 514O_6,10_—at a molar ratio of 35% lipidoid/16% DOPE/46.5% cholesterol/2.5% PEG-lipid and administered to C57BL/6 mice (mRNA at a dose of 0.5 mg/kg) (*n* = 3 mice per group). Three hours later, mice were injected with d-luciferin, euthanized, and dissected for ex vivo luminescence imaging using in vivo imaging system (IVIS). The left panels depict representative IVIS images of key organs, the middle panels quantify mLuc expression, the right panels illustrate the percentage of protein expression occurring per organ. Compared to intravenous (IV) delivery, intraperitoneal (IP) administration increased mRNA delivery for all formulations. Error bars represent SEM. Student’s *t* tests were used to compare intravenous and intraperitoneal delivery for each organ. ****P < 0.005 and ****P < 0.001. Data represent three biological replicates. Statistics are color-coded according to organ. Panc, pancreas; Sp, spleen.

Achieving robust and selective non-viral mRNA delivery to target tissues outside the liver and spleen remains an unresolved challenge in gene therapy. We rationalized that intraperitoneal injection might facilitate mRNA delivery to the pancreas. To test this, we formulated three LNPs, each containing a unique ionizable lipidoid: 306O_i10_, 200O_i10_, or 514O_6,10_ (structures shown in fig. S1). We chose these materials because they facilitate robust mRNA delivery and produce different protein expression profiles (Fig. 1) (*22*, *29*, *35*). We formulated LNPs containing mRNA encoding for firefly luciferase (mLuc) and delivered them to C57BL/6 mice at a dose of 0.5 mg/kg using either intravenous or intraperitoneal administration. Three hours later, we euthanized the mice and analyzed their organs for luminescent signal ex vivo using an in vivo imaging system (IVIS). For all three of these materials, intraperitoneal injection enhanced mRNA delivery to the pancreas in terms of efficacy and specificity compared to intravenous delivery (Fig. 1). For example, 306O_i10_ produced the highest levels of protein expression in the pancreas, while the specificity for the pancreas with 514O_6,10_ increased from 2% (intravenous) to 52% (intraperitoneal). These results indicate that non-viral gene delivery to the pancreas is possible and that intraperitoneal injections facilitate improved delivery and specificity compared to intravenous administration. Although relatively uncommon, intraperitoneal injections are routinely used in the clinic for delivering chemotherapy to peritoneal cancers (*49*). Therefore, this strategy may be a viable option to treat pancreatic diseases associated with high morbidity and mortality such as cancer or diabetes. Furthermore, gene delivery to the pancreas has thus far predominantly necessitated infusing viral vectors through the pancreatic duct. Intraperitoneal injection, in comparison, offers decreased risk to the patient. Therefore, using intraperitoneal injection for LNP administration is likely a clinically viable strategy for severe pancreatic diseases.

### Cationic helper lipids improve delivery specificity for the pancreas

**Fig. 2.**
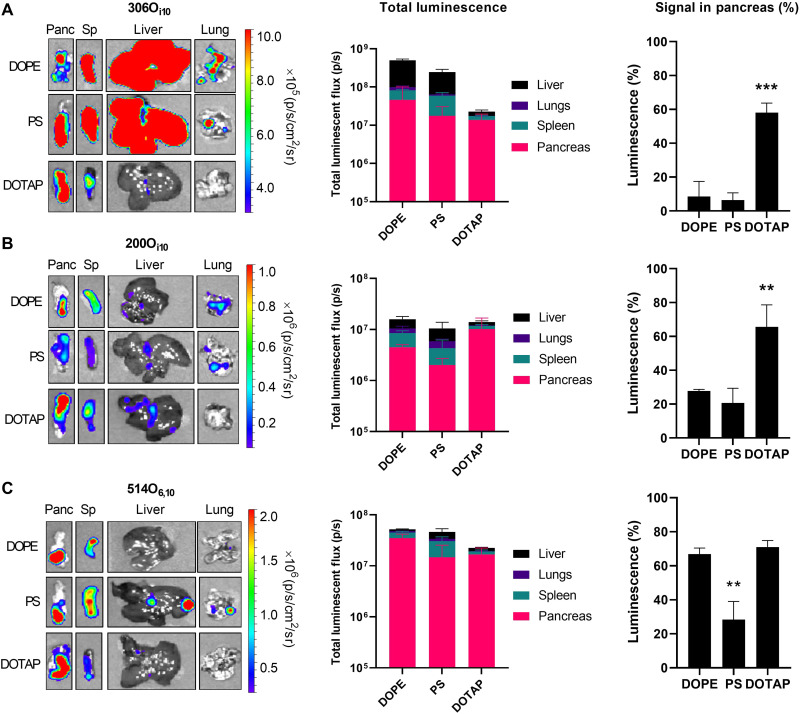
The helper lipid DOTAP improves specificity for the pancreas by decreasing off-target protein expression. LNPs containing mLuc were formulated using each of three different lipidoids—(**A**) 306O_i10_, (**B**) 200O_i10_, or (**C**) 514O_6,10_—in a molar ratio of 35% lipidoid/40% helper lipid/22.5% cholesterol/2.5% PEG-lipid and administered to C57BL/6 mice (mRNA at a dose of 0.5 mg/kg) (*n* = 3 mice per group). Three hours later, mice were injected with d-luciferin, euthanized, and dissected for ex vivo luminescence imaging using IVIS. The left panels depict representative IVIS images of key organs, the middle panels quantify mLuc expression, the right panels illustrate the percentage of protein expression occurring in the pancreas. Error bars represent SEM. Analysis of variance (ANOVA) with post hoc Tukey-Kramer was used to compare the fraction of total signal that occurred within the pancreas across helper lipids. **P < 0.01 and ***P < 0.005.

Having demonstrated that intraperitoneal administration facilitates mRNA delivery to the pancreas, we next optimized our LNP formulation to maximize pancreatic protein expression while minimizing off-target delivery to the liver and spleen. Cheng *et al.* (*40*) demonstrated that formulating LNPs with helper lipids of different charges enabled tissue-specific mRNA delivery following intravenous injection. On the basis of this, we hypothesized that modulating helper lipid chemistry and charge would facilitate potent and specific mRNA delivery to the pancreas. To examine this hypothesis, we used the three previous ionizable lipids to create nine additional LNP formulations. Each formulation contained a differently charged helper lipid constituting 40% of the total amount of lipid (structures shown in fig. S2). Specifically, we formulated LNPs with either the zwitterionic helper lipid 1,2-dioleoyl-*sn*-glycero-3-phosphoethanolamine (DOPE), the anionic helper lipid l-α-phosphatidylserine (PS), or the cationic helper lipid 1,2-dioleoyl-3-trimethylammonium-propane (DOTAP). LNPs containing these helper lipids and mLuc were delivered to C57BL/6 mice at a dose of 0.5 mg/kg via intraperitoneal injection. Three hours later, the mice were euthanized, and their organs were analyzed for luminescent signal ex vivo using IVIS (Fig. 2).

For each lipidoid, the three different helper lipids produced roughly equivalent signal in the pancreas (Fig. 2). However, the cationic helper lipid DOTAP most effectively reduced off-target luciferase expression in the liver and spleen for all lipidoids. To confirm that this result depends on the presence of ionizable lipid rather than the cationic property of DOTAP alone, we compared the performance of 306O_i10_ and DOTAP LNPs with that of DOTAP LNPs lacking ionizable lipid. As expected, LNPs containing ionizable lipid induced two orders of magnitude greater protein expression than LNPs containing DOTAP alone (fig. S3). We next determined whether our findings are extendable to other cationic helper lipids. We tested 306O_i10_ LNPs containing mLuc and formulated with DOTAP, 1,2-dioleoyl-sn-glycero-3-ethylphosphocholine (EPC), or dimethyldioctadecylammonium bromide (DDAB). The resultant protein expression was similar across cationic lipids in both magnitude and specificity for the pancreas (fig. S4).

Among DOTAP-containing LNPs, the improvement in specificity for the pancreas was most prominent for the lipidoid 306O_i10_. While each of the three helper lipids produced ~2 × 10^7^ to 5 × 10^7^ photons/s (p/s) luminescent flux in the pancreas, this constituted ~10% of the total signal for the helper lipids DOPE and PS and 60% of the total signal for DOTAP, with the remaining signal primarily occurring in the liver and spleen (Fig. 2A). While LNPs formulated with 200O_i10_ produced the least overall luminescent signal, DOTAP again significantly improved the percentage of total signal appearing in the pancreas. For 514O_6,10_, both DOPE and DOTAP enabled >60% specificity for the pancreas. However, the amount of total signal including off-target signal in the liver and spleen is greater for DOPE than DOTAP. Depending on the application, it may be preferable to minimize off-target delivery at the cost of slight potency loss in the target tissue. As a hypothetical example, in an mRNA therapy engineered to deliver suicide genes to pancreatic cancer cells, it would be better to eliminate as much off-target delivery as possible even at the cost of additional delivery to the pancreas (*26*). For this reason, we proceeded with 306O_i10_ LNPs formulated with 40% DOTAP for the remainder of our studies.

### Pancreatic protein expression persists for days and correlates with mRNA biodistribution

One advantage of mRNA therapeutics is that the resultant protein expression is transient, with translation half-lives ranging from ~7 to 30 hours depending on the route of administration (*30*). Previous work demonstrated that intravenous administration produces the shortest translation half-life (~7 hours), while intramuscular and intradermal delivery can extend mRNA translation, with half-lives of 20 and 30 hours, respectively (*30*). Therefore, we sought to understand protein expression kinetics following intraperitoneal administration of pancreas-tropic LNPs.

**Fig. 3.**
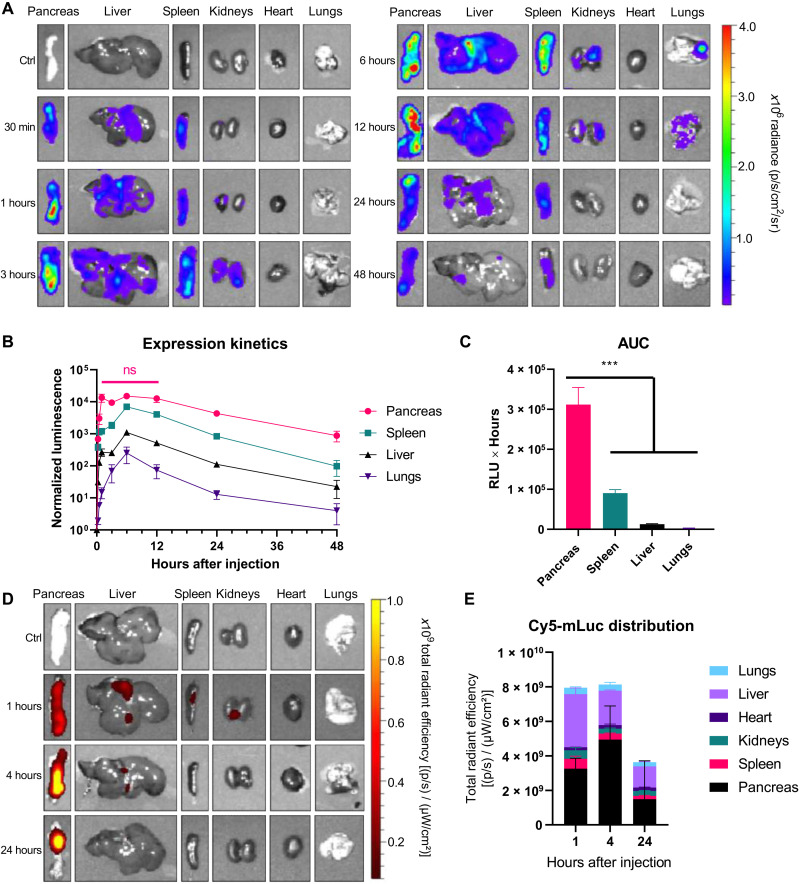
Protein expression in the pancreas persists for at least 48 hours following mRNA LNP injection. LNPs containing (**A** to **C**) mLuc or (**D** and **E**) Cy5-mLuc were formulated using the lipidoid 306O_i10_ in a molar ratio of 35% lipidoid/40% DOTAP/22.5% cholesterol/2.5% PEG-lipid and administered to C57BL/6 mice (mRNA at a dose of 0.5 mg/kg) (*n* = 3 mice per group). Unlabeled mLuc (A to C) allows the detection of functional translated protein, while Cy5-mLuc (D and E) is used to detect the presence of mRNA molecules. At the indicated times, mice were injected with d-luciferin, euthanized, and dissected for ex vivo luminescence (A to C) or fluorescence (D and E) imaging using IVIS. Error bars represent SEM. In (C), ANOVA with post hoc Tukey-Kramer was used to compare the area under the curve (AUC) for each organ; ***P < 0.005. Ctrl, control.

In these studies, mice received intraperitoneal injections of mRNA (0.5 mg/kg) and were euthanized for IVIS imaging at time points between 15 min and 48 hours. Representative images (Fig. 3A) and quantified luminescence (Fig. 3B) show that protein expression peaked at ~6 hours in all organs analyzed and persisted through at least 48 hours. In the pancreas, luminescence persisted at its maximum from 1 to 12 hours (Fig. 3B). Area under the curve (AUC) analysis shows that total protein expression in the pancreas was 3.5-fold greater than that in the spleen over a 48-hour period, 24-fold greater than that in the liver, and 137-fold greater than that in the lungs (Fig. 3C). How these findings extend to other proteins will depend on how the mRNA sequence interacts with regulatory elements in pancreatic cells and on the half-life of the protein in question. For example, the half-life of firefly luciferase is approximately 3 to 4 hours in mammalian cells (*50*), so the persistence of other proteins will likely differ from luciferase.

To further characterize the pancreatic specificity of this delivery system, we assessed mRNA biodistribution kinetics, as mRNA biodistribution and protein expression do not always correlate (*29*). In this experiment, we intraperitoneal-injected LNPs containing Cyanine 5 (Cy5)-mLuc at a dose of 0.5 mg/kg assessed Cy5 fluorescence in dissected organs using IVIS as a function of time. We found that pancreatic Cy5-mRNA signal was greatest throughout the experiment and peaked ~4 hours after injection (Fig. 3, D and E). There was also some accumulation in the liver, which peaked ~1 hour after injection and decreases thereafter. This is consistent with the biodistribution kinetics from peripheral blood (fig. S5) and with data on apolipoprotein E-mediated delivery of intravenous-injected LNPs to hepatocytes (*51*). Despite the observation that luciferase expression occurred in the spleen, we did not detect substantial Cy5-mLuc distribution to the spleen. Together with previous results indicating that only a small fraction of monocytic splenocytes translates LNP-formulated mRNA (*29*), our findings indicate that the nucleoside-modified mRNA used in these experiments does not require high levels of splenic accumulation spleen to induce high levels of protein expression.

### A single dose of LNPs can simultaneously deliver multiple mRNAs to the pancreas

While delivering one mRNA may be sufficient for single-protein replacement therapies, more advanced applications such as mRNA-based immunotherapies or regenerative medicine may require the delivery of multiple mRNAs simultaneously. For example, pancreatic cancer is a tremendously complex disease that would likely necessitate the delivery of multiple protein-coding mRNAs to activate tumor suppressive signaling and repress tumorigenic pathways (*52*, *53*). In the case of autoimmune diabetes, insulin-producing β cells can be regenerated by delivering a cocktail of β cell–specific transcription factors to endocrine or exocrine pancreatic cells (*43*, *54*, *55*). In addition, gene editing applications require the simultaneous delivery of editing enzymes and guide RNAs (*10*). Therefore, we next asked whether our pancreatic mRNA delivery strategy is sufficiently robust to achieve this.

**Fig. 4.**
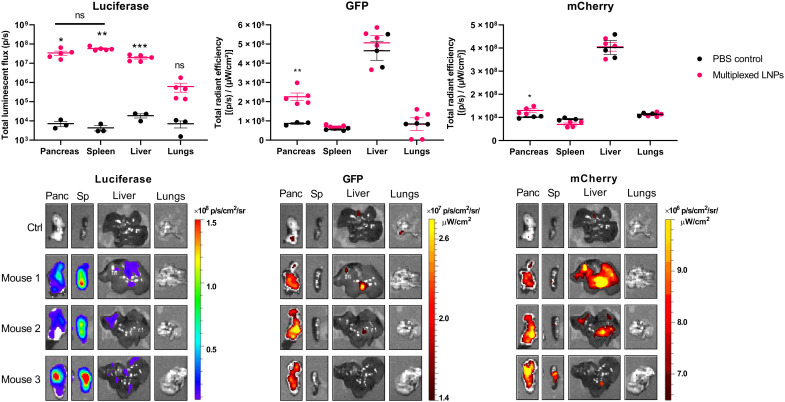
Multiple mRNAs can be simultaneously delivered to the pancreas. LNPs containing mRNA encoding firefly luciferase, GFP, and mCherry were formulated using the lipidoid 306O_i10_ in a molar ratio of 35% lipidoid/40% DOTAP/22.5% cholesterol/2.5% PEG-lipid and administered to C57BL/6 mice (mRNA at a dose of 0.6 mg/kg) (0.2 mg/kg each mRNA, *n* = 5 mice per group). Six hours later, mice were injected with d-luciferin, euthanized, and dissected for ex vivo luminescence and fluorescence imaging using IVIS. Error bars represent SEM. Student’s *t* test was used to compare signal from control untreated mice versus mice receiving multiplexed LNPs for each organ. *P < 0.05, **P < 0.01, and ***P < 0.005. ns, not significant.

To test this, we formulated LNPs with three reporter protein-encoding mRNAs: firefly luciferase, green fluorescent protein (GFP), and mCherry. LNPs were formulated with equal amounts of each mRNA and injected intraperitoneally into mice (mRNA at a dose of 0.6 mg/kg). Six hours later, mice were euthanized, and organs were imaged ex vivo using IVIS to quantify the resultant protein expression. As shown in Fig. 4, significant luminescence was detected in the pancreas, spleen, and liver, while significant GFP and mCherry fluorescence was detected only in the pancreas.

### LNPs transfect primarily pancreatic β cells

Next, we determined which cell types in the pancreas were transfected with intraperitoneal-injected mRNA LNPs. The pancreas performs both exocrine and endocrine functions (*56*). Acinar tissue, which makes up about 98% of the pancreas, secretes enzymes that aid in digestion. The islets of Langerhans account for ~1 to 2% of the pancreas and are responsible for maintaining glucose homeostasis. Pancreatic islets comprise multiple cell types, including α, β, δ, ε, and pancreatic polypeptide cells. β cells produce insulin and constitute ~70% of the total islet cells, while α cells secrete glucagon and make up 20% of the islets (*57*). To determine which cell types undergo transfection, we performed immunohistochemical analysis on fixed/frozen mouse pancreas sections to probe for luciferase protein expression in mice that were treated with intraperitoneal-injected mRNA LNPs (0.5 mg/kg). Mice receiving phosphate-buffered saline (PBS) injections were used as a control.

**Fig. 5.**
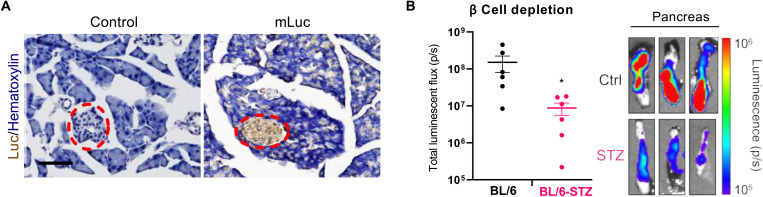
LNPs transfect primarily pancreatic islets. (**A**) LNPs containing mLuc were injected intraperitoneally into mice [mRNA (0.5 mg/kg)]. Six hours later, mice were euthanized, and pancreata were fixed, frozen, and sectioned onto slides. Slides were stained for luciferase by immunohistochemistry and counterstained with hematoxylin. Islets are within the red dashed ovals. Scale bar, 100 μm. Additional images are shown in fig. S6. (**B**) Depleting β cells in C57BL/6 mice with STZ reduces total mLuc delivery to the pancreas. Error bars represent SEM; *P = 0.036 by Student’s *t* test. The images on the right represent IVIS luminescence images of three independent replicates of control or STZ mice treated with mLuc LNPs.

Histology revealed that most luciferase expression occurs within the islets, with less intense luciferase staining appearing throughout the acinar tissue (Fig. 5A). Additional replicates are shown in fig. S6. These results were unexpected, as islets constitute a small fraction of the total pancreatic tissue and are surrounded by a thin capsule of fibrous connective tissue (*58*). This capsule presents an additional barrier against LNP delivery to the islets.

To confirm these results, we used a mouse model of β cell depletion to interrogate whether depleting β cells, which make up most of the islet, reduces the total mRNA delivery to the pancreas. In these studies, we used C57BL/6 mice that had been treated with streptozotocin (STZ), which selectively depletes β cells (*59*). This is commonly used as a model of type 1 diabetes, in which β cells are destroyed due to autoimmunity. Depleting β cells with STZ significantly decreased luciferase mRNA delivery to pancreas, as was evidenced by a 94% reduction in pancreatic luminescence observed in STZ mice compared to controls (Fig. 5B). This is remarkable, in our opinion, considering that β cells constitute only ~1% of the total cells in the pancreas but account for nearly all of the protein expression resulting from transfection of normal pancreata.

### Peritoneal macrophages contribute to pancreatic mRNA delivery

**Fig. 6.**
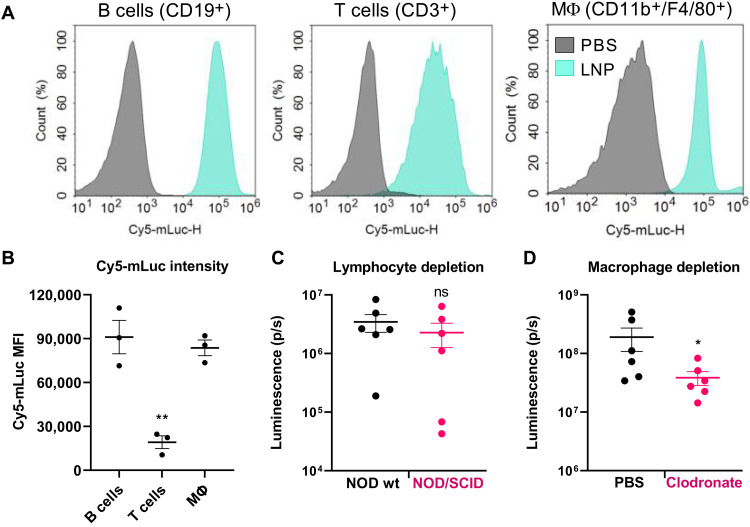
Peritoneal macrophages contribute to pancreatic mRNA delivery following intraperitoneal administration. (**A**) Mice were injected intraperitoneally with LNPs containing Cy5-mLuc (0.5 mg/kg). Immediately after injection, mice were euthanized, and peritoneal wash was collected for flow cytometry analysis. Cy5-mLuc is associated with B cells, T cells, and CD11b^+^/F4/80^+^ macrophages. (**B**) Median fluorescence intensity (MFI) of Cy5-mLuc in B cells, T cells, and CD11b^+^/F4/80^+^ macrophages. (**C**) Lymphocyte trafficking does not facilitate mRNA delivery to the pancreas. Wild-type (wt) NOD mice or NOD/SCID mice that lack mature lymphocytes were intraperitoneally injected with LNPs containing mLuc or Cy5-mLuc (0.5 mg/kg) and euthanized for ex vivo IVIS analysis 3 hours later. There were no differences in either Luc expression or Cy5 distribution to the pancreas resulting from lymphocyte depletion. (**D**) Mice were injected intraperitoneally with PBS (control) or clodronate liposomes to deplete macrophages. Forty-eight hours later, mice were injected intraperitoneally with mLuc-LNPs, and luminescence in the pancreas was quantified by IVIS. Transfection efficacy decreased in macrophage-depleted mice. Error bars represent SEM. *P < 0.05 and **P < 0.005 by one-way ANOVA with post hoc Tukey’s test.

We next determined the mechanism by which LNPs reach the pancreas following intraperitoneal delivery. While we initially speculated that this might occur due to the organ’s location in the peritoneal cavity, which would not explain the reduced protein expression in the liver and spleen. Therefore, we examined the cellular environment of the peritoneum following intraperitoneal injection of LNPs. Because the peritoneum is a rich source of immune cells, we hypothesized that these cells were interacting with the LNPs. To investigate this, we delivered intraperitoneally LNPs formulated with Cy5-mLuc to mice at a dose of 0.5 mg/kg. We euthanized the mice immediately, harvested the peritoneal immune cells, and analyzed them by flow cytometry. This experiment showed that LNPs containing Cy5-mLuc were immediately bound to or internalized by nearly all T cells, B cells, and macrophages (Fig. 6A). Furthermore, LNP uptake by B cells and macrophages was significantly greater than that of T cells (Fig. 6B).

Intrigued by this, we next asked whether these cell populations were contributing to pancreatic mRNA delivery. To determine whether lymphocytes are necessary for pancreatic mRNA delivery, we used a nonobese (NOD)/severe combined immunodeficient (SCID) mouse model, which lacks mature B cells and T cells. Therefore, if these cells are essential for pancreatic mRNA delivery, we would expect decreased pancreatic protein expression in NOD/SCID mice relative to wild-type NOD controls. We treated NOD or NOD/SCID mice with mLuc LNPs delivered intraperitoneally at 0.5 mg/kg and found that there was no significant decrease in pancreatic luminescence in NOD/SCID mice relative to the wild-type control (Fig. 6C). Therefore, we concluded that lymphocytes do not contribute to the transfection process.

To test for macrophage involvement, we used clodronate liposomes according to the manufacturer’s protocol to deplete macrophages 48 hours before LNPs delivery. Clodronate depletion significantly decreased luciferase signal in the pancreas (Fig. 6D) relative to mice receiving control PBS injections, suggesting that macrophages are critical for mRNA delivery to the pancreas following intraperitoneal injection. We postulated that two possible mechanisms could underly macrophage-mediated pancreatic mRNA delivery. First, transfected macrophages may secrete EVs (*60*) containing either the intact LNPs or undamaged mRNA from the peritoneal cavity that then enter the pancreas directly or via lymphatic circulation. We considered the possibility that EVs contain translated protein; however, the mRNA biodistribution results in Fig. 3D indicate that mRNA does reach the pancreas. Alternatively, macrophages may migrate directly into pancreatic tissue and horizontally transfer intact LNPs or mRNA to cells within the pancreas. There is precedence for this potential mechanism, given previous research showing that lipoplexes containing siRNA or DNA are taken up by macrophages and subsequently transferred to cancer cells (*60*).

### EVs secreted by LNP-treated macrophages induce protein expression in pancreatic islet cells

**Fig. 7.**
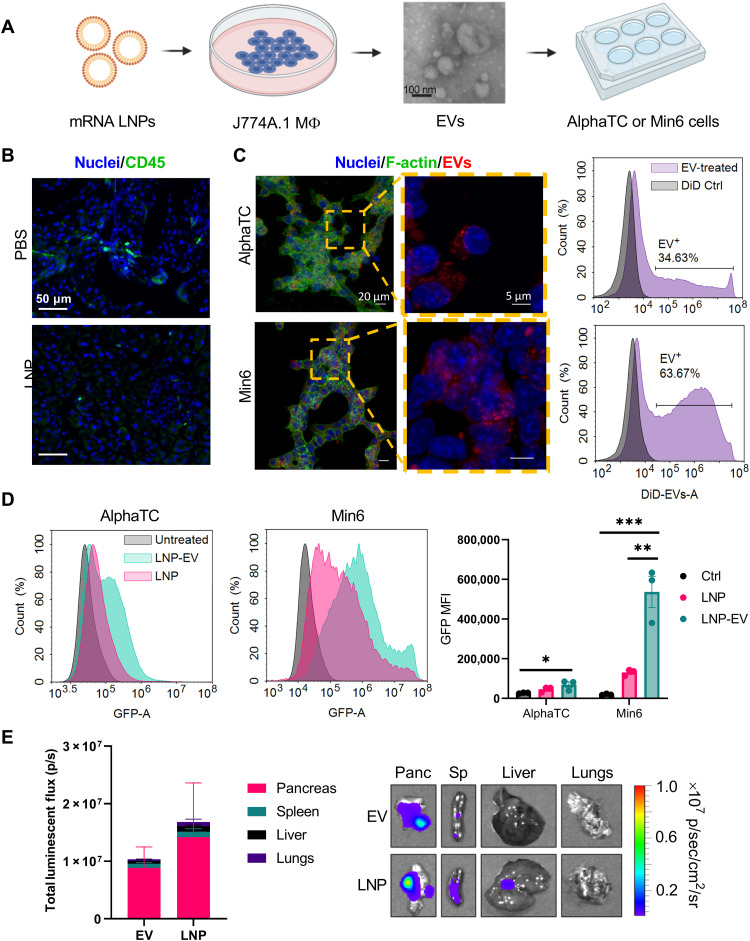
EVs isolated from LNP-treated macrophages transfect pancreatic islet cells. (**A**) To determine the role of EVs in pancreatic mRNA delivery, J774A.1 macrophages were treated with mRNA LNPs. 48 hours later, EVs were isolated from the supernatant by size exclusion chromatography (SEC) and characterized by electron microscopy (additional characterization in the Supplementary Materials). Purified EVs (20 μg) were then added to AlphaTC Clone 9 α cells or Min6 β cells in culture. (**B**) CD45 staining of mouse pancreas sections indicated no immune cell infiltration following LNP injection [mLuc (0.5 mg/kg), 3 hours after injection]. Additional replicates are shown in fig. S7. (**C**) Uptake of DiD-labeled EVs by AlphaTC and Min6 cells was assessed by confocal microscopy and flow cytometry. In the flow cytometry histograms, the control sample represents a control for residual DiD after exosome staining. (**D**) By flow cytometry, EVs from J774A.1 macrophages treated with mGFP mRNA-LNPs induce greater GFP expression in AlphaTC and Min6 cells than LNPs. AlphaTC and Min6 cells received the same LNP dose as the J774A.1 macrophages. (**E**) Peritoneal macrophages were isolated from mice and transfected with LNPs ex vivo. EVs were then isolated from the macrophages and injected intraperitoneally into mice, inducing pancreatic protein expression. Error bars represent SEM. *P < 0.05, **P < 0.01, and ***P < 0.001 by one-way ANOVA with post hoc Tukey’s test.

Next, we tested these hypotheses to determine how macrophages mediate pancreatic mRNA delivery. An influx of macrophages into the pancreas might suggest an unwanted inflammatory response and is likely undesirable. To rule out this possibility, we assessed the presence of CD45^+^ immune cells in the pancreata of mice treated with PBS or LNPs (containing mLuc) by immunofluorescence staining and confocal microscopy. As shown in Fig. 7B and fig. S7 (additional replicates), we did not observe an increase in CD45^+^ cells in LNP-treated pancreas sections, indicating that immune cells are not infiltrating the pancreas following LNP administration.

To interrogate the possibility of macrophage EV-mediated gene transfer, we used an in vitro model in which EVs can be collected and analyzed (Fig. 7A). In these experiments, J774A.1 mouse macrophages were treated with mRNA LNPs. Forty-eight hours later, the supernatant was collected, and EVs were purified using size exclusion chromatography (SEC) (*61*–*63*). EVs were ~125 nm in diameter by nanoparticle tracking analysis (NTA) and transmission electron microscopy and were positive for endosomal markers via Western blot (fig. S8). Purified EVs were then delivered to AlphaTC Clone 9 mouse pancreatic α cells or Min6 mouse pancreatic β cells. To visualize the uptake of EVs by islet cell lines, EVs were stained with 1,1'-dioctadecyl-3,3,3',3'-tetramethylindodicarbocyanine, 4-chlorobenzenesulfonate (DiD) before transfection. EVs were visualized in both cell lines by confocal microscopy (Fig. 7C). Furthermore, flow cytometry analysis revealed that Min6 β cells take up EVs to a greater extent than AlphaTC α cells.

We next assessed whether EV uptake by AlphaTC α and Min6 β cells induces protein expression. The cells exhibited greater GFP fluorescence after incubation with LNP-EVs containing mRNA encoding green fluorescent protein (mGFP) than cells treated with LNPs directly (Fig. 7D). While this held true for both cell lines, Min6 β cells yielded the greatest enhancement in GFP expression with EVs, with the resultant fluorescence increasing ~7.5-fold compared to Min6 cells treated with LNPs. These results are consistent with what has been previously described for small noncoding RNAs (*64*, *65*).

Last, we sought to determine whether these findings would translate to in vivo delivery. We isolated and cultured primary peritoneal macrophages from C57BL/6 mice and subsequently treated them with mRNA-LNPs. Seventy-two hours later, EVs were isolated using SEC, and their concentration was determined using NTA. To compare protein expression resulting from LNP-EVs with that produced by LNPs directly, we normalized dosing by particle count. By NTA, 100 μl of LNPs formulated with 10 μg of mLuc contained 1.5 × 10^11^ LNPs. Therefore, we assessed luciferase expression resulting from intraperitoneal injections of either 1.5 × 10^11^ LNPs or 1.5 × 10^11^ LNP-EVs. IVIS analysis of dissected organs revealed that LNPs and LNP-EVs produced similar levels of luciferase expression in the pancreas (Fig. 7E).

### Intraperitoneal delivery of mRNA-LNPs does not induce cytokine release syndrome or tissue damage

**Fig. 8.**
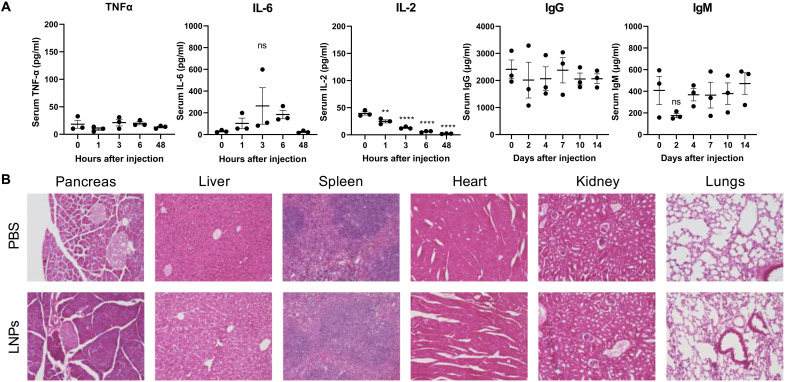
Pancreas-targeting LNPs are tolerated by mice. In these studies, mice were treated with mLuc mRNA-LNPs (0.5 mg/kg), and serum or tissue samples were collected for analysis at the indicated time points. (**A**) By enzyme-linked immunosorbent assay, there are no significant increases in systemic inflammatory cytokines, IgG, or IgM after LNP treatment. (**B**) H&E staining indicates no signs of tissue damage 8 days after mRNA-LNP treatment. Images were collected at ×20 magnification.

In addition to efficacy, safety and immunogenicity are other critical factors that affect a therapeutic’s performance in the clinic. Unchecked immune responses can result in severe adverse events, including tissue inflammation, anaphylactic reactions, and organ damage (*66*). To evaluate the systemic immunogenicity of our pancreas-targeting mRNA-LNP formulation, we measured the levels of the proinflammatory cytokines, tumor necrosis factor–α (TNF-α), interleukin-6 (IL-6), and IL-2, as well as the immunoglobulins immunoglobulin G (IgG) and IgM. We found no increase in TNF-α serum levels and a slight but insignificant and transient increase in IL-6 serum levels in treated mice for 48 hours after mRNA-LNP injection (Fig. 8A), suggesting minimal activation of patrolling innate immune cells, such as monocytes and macrophages. Furthermore, we observed decreasing IL-2 levels after injection (Fig. 8A). IL-2 is a marker for T cell proliferation (*67*), and decreasing IL-2 levels suggest that mRNA-LNPs do not evoke an acute T cell–mediated response. Next, we observed no changes in total IgG levels over 2 weeks (Fig. 8A). We also saw an insignificant decrease in IgM levels immediately after mRNA-LNP injection (Fig. 8A), which may be attributed to naïve IgM antibodies adsorbing onto the surface of mRNA-LNPs as part of the protein corona (*68*). IgM returned to baseline levels 2 weeks after injection (Fig. 8A), implying the absence of an IgM-mediated humoral response. Together, these data indicate that mRNA-LNPs do not evoke a potent systemic immune response.

Qualitative analysis of organs stained with hematoxylin and eosin (H&E) revealed no evidence of tissue damage 8 days after mRNA-LNP treatment. Slight changes in H&E staining intensity may suggest remnants of a mild inflammatory response expected from robust mRNA delivery. In the pancreas, the darker staining may indicate this mild inflammation. However, the pancreas tissue appears healthy with intact islets identified at ×20 magnification. The liver, spleen, heart, kidney, and lung tissue samples appear similar to PBS-treated controls, indicating no adverse results of mRNA-LNP treatment in these tissues.

## DISCUSSION

To date, biological drug delivery barriers have hindered the successful development of non-viral gene therapies for pancreatic diseases. Here, we describe a nucleoside-modified mRNA-LNP platform to achieve this. While intravenous-administered nanoparticles have deliver siRNA and mRNA (*69*) to pancreatic cancer models, nearly all these studies used subcutaneous or orthotopic tumor xenograft models (*70*, *71*), which do not recapitulate native blood circulation to the pancreas. In addition, tumor architecture provides advantages for gene delivery that are not present in other pancreatic diseases such as pancreatitis and diabetes (*72*, *73*). As a result, alternative routes of administration are needed to deliver genes to the pancreas.

Three primary routes of administration have been investigated for gene delivery to the pancreas: intravenous, intraperitoneal, and infusion through the pancreatic duct (*74*). Intravenous delivery of adeno-associated virus (AAV)–based gene therapies weakly transduces pancreatic acinar tissue and requires blocking circulation to the liver by ligating the bile duct (*74*, *75*). Intraperitoneal delivery of AAVs leads to protein expression within islets, while maximum gene delivery was achieved using intraductal infusion (*74*). Intraductal infusion has been adopted as a favorable strategy for pancreatic gene delivery in preclinical testing (*43*, *76*–*79*). While this is effective and clinically feasible, a less invasive delivery route would be preferred. Moreover, while viruses are extremely efficient cellular transducers, they risk integration into the host genome and are limited by their immunogenicity (*44*, *80*). The nucleoside-modified mRNA-LNP platform presented here can overcome these limitations.

We report that intraperitoneal delivery of LNPs promotes pancreatic mRNA delivery and subsequent protein expression (Fig. 1). While LNP absorption following intraperitoneal delivery remains poorly understood, reports on intraperitoneal-delivered liposomes indicate that the route of absorption depends on size, lipid composition, and surface PEG density (*47*, *48*, *81*). Nanoparticles are precluded from entering the bloodstream from the peritoneal cavity by mesothelial and endothelial barriers. Instead, nanoparticles are more likely to drain into lymphatic vessels via the diaphragmatic lymphatics (*81*). However, cationic liposomes and nanoparticles have shown greater retention within the peritoneal cavity due to interactions with the negatively charged mesothelium (*47*). This may account for the small percentage of the injected LNP dose detectable in the bloodstream (fig. S3) and decreased delivery to the liver and spleen (Fig. 2).

LNPs containing cationic lipids may benefit pancreatic mRNA delivery in several ways. First, cationic phospholipids direct LNP-mediated mRNA delivery away from the liver and spleen upon intravenous injection (*40*, *42*). Furthermore, physicochemical interactions may account for the prominent delivery of mRNA to pancreatic islets. Islets are separated from the surrounding exocrine tissue by the peri-islet membrane—a thin basement membrane that consists of negatively charged extracellular matrix components including laminins, collagen IV, and perlecan (*82*). Therefore, LNPs containing cationic lipids may interact favorably with the peri-islet membrane, leading to mRNA delivery within islets.

In addition to these physicochemical interactions, we showed that LNPs interact with B cells, T cells, and macrophages in the peritoneal cavity, while only macrophages contribute appreciably to pancreatic mRNA delivery (Fig. 6). Because we did not observe immune cells infiltrating the pancreas (Fig. 7B), we postulate that macrophages exposed to LNPs secrete EVs that transfer their cargo to cells within the pancreas. There is precedent for this proposed mechanism, as macrophage-mediated gene transfer has been previously reported as an efficient strategy to deliver siRNA lipoplexes to cancer cells (*60*), and the use of EVs is of widespread interest for gene and drug delivery (*83*–*85*). Specifically, M2 anti-inflammatory macrophages, the phenotype exhibited by peritoneal macrophages (*86*), are particularly efficient at horizontal gene transfer due to their enhanced EV secretion via Rab27 signaling (*60*). EVs secreted by macrophages exposed to LNPs may contain either mRNA or translated protein. Our biodistribution results indicate that intraperitoneal-injected LNPs deliver Cy5-mRNA to the pancreas, but future research is needed to determine whether these EVs contain intact LNPs or unpackaged mRNA cargo. Furthermore, we have previously reported that nucleoside-modified mRNA, used in the present study, preferentially increases protein translation in macrophages and other monocytic cells over other cell types (*29*), suggesting that macrophages may produce high levels of protein that is, in turn, secreted via EVs.

We were excited to find that EVs derived from LNP-treated macrophages transfected pancreatic cells at least and LNPs, both in vitro and in vivo (Fig. 7, D and E). The advantages of EV-mediated delivery have been reported for EVs loaded with small noncoding RNAs but have not been studied extensively with exogenous mRNA. One study reported similar findings that EVs secreted from LNP-treated NSC-34 motor neuron-like cells effectively deliver silencing RNAs to the liver and intestines of mice at 10-fold lower doses than LNPs (*64*). This is supported by recent work demonstrating that two orders of magnitude less sgRNA is required to achieve significant gene delivery with EVs compared with LNPs (*65*). We unexpectedly observed this trend in our studies because the passive packaging of RNA into EVs is a rare event not expected to sufficiently induce gene transfer (*65*), with serum-derived EVs exhibiting a capacity of one RNA molecule or less (*87*). It is also important to consider how lipids from LNPs might interact with EVs. Because lipids commonly used in LNPs often have fusogenic properties, it is likely that some or all of these components become incorporated into the secreted EV (*88*). Continued research is needed to elucidate how exogenous mRNAs, and lipids are packaged into EVs and inform new strategies to maximize their transfer to target cells.

LNP-mediated mRNA delivery to the pancreas could catalyze the development of unprecedented therapeutics for incurable diseases such as pancreatic cancer or diabetes, which affects roughly 10% of the U.S. population (*89*). For example, delivering mRNA encoding hepatocyte growth factor (HGF) to β cells could provide an effective treatment strategy for type 1 or type 2 diabetes. Previous studies show that HGF induced by transgenic overexpression or by recombinant AAV vectors can increase insulin production and β cell proliferation to confer resistance against the diabetogenic effects of STZ in mice (*76*, *90*). Similarly, expressing the transcription factors Pdx1 and MafA in neighboring α cells can convert these cells into functional, insulin-producing β cells that appear resistant to autoimmune destruction in models of type 1 diabetes (*43*). Other reports demonstrate that adding the gene Ngn3 to this cocktail can transdifferentiate pancreatic exocrine cells into β-like cells as well (*54*, *55*). Moreover, many genes are known to be dysregulated in pancreatic cancer. For instance, KRAS overexpression and loss of p53 are associated with poor pancreatic cancer outcomes and could be reversed using mRNA LNPs (*91*, *92*). Notably, these approaches would leverage the multiplexed delivery capabilities that we demonstrate in the present study (Fig. 4).

Through this work, we have developed a strategy to achieve non-viral gene delivery to the pancreas using nucleoside-modified mRNA-LNPs. We have further identified EV secretion and gene transfer by macrophages as a mechanism by which LNPs can deliver mRNA to challenging organ targets. Moving forward, there are key differences in pancreas structure between mice and humans that should be investigated, including differences in islet composition, acinar and ductal tissue organization, and vascularization (*93*). Together, these data show that LNPs facilitate non-viral protein expression in pancreatic islets and have the potential to transform the therapeutic landscape for deadly, intractable diseases of the pancreas.

## MATERIALS AND METHODS

### Materials

The amines 3,3′-diamino-*N*-methyldipropylamine (306) was purchased from Sigma-Aldrich (St. Louis, MO), and N1-{2-[4-(2-aminoethyl)piperazin-1-yl]ethyl}ethane-1,2-diamine (200) were acquired from Enamine (Princeton, NJ). The tail isodecyl acrylate (O_**i10**_) was purchased from Sartomer (Colombes, France). Cholesterol was sourced from Sigma-Aldrich. The lipids DOPE, PS, DOTAP, and C14-PEG2000 were purchased from Avanti Polar Lipids (Alabaster, AL). mRNAs were obtained from TriLink Biotechnologies (San Diego, CA) including the 5-methoxyuridine base modification or synthesized by in vitro transcription using the MEGAscript T7 Transcription Kit (Thermo Fisher Scientific, Waltham, MA). XenoLight d-luciferin potassium salt was procured from PerkinElmer (Waltham, MA). IL-2, IL-6, and TNF-α mouse enzyme-linked immunosorbent assay (ELISA) kits were purchased from Thermo Fisher Scientific (Waltham, MA). Mouse IgG and IgM ELISA kits were obtained from Abcam (Cambridge, MA).

### In vitro mRNA transcription

mRNAs were synthesized as previously described (*94*, *95*). Briefly, linearized plasmids encoding firefly luciferase, enhanced GFP, and mCherry were transcribed using the MEGAscript T7 Transcription Kit (Ambion) with cotranscriptional capping with CleanCap reagent AG (TriLink Biotechnologies). One-methylpseudouridine (m1Ψ)-5′-triphosphate (TriLink) instead of uridine 5′-triphosphate was used to generate modified nucleoside-containing mRNA. Double-stranded RNA (dsRNA) contaminants were removed by cellulose purification as described (*96*). Briefly, 700 μg of crude mRNA was loaded into a spin column containing 700 μl of 16% cellulose (w/v). Columns were incubated at room temperature with constant shaking for 25 min. dsRNA-free mRNA was collected in the flow-through and then purified a second time. All mRNAs were analyzed by electrophoresis using agarose gels and confirmed to be free of dsRNA contaminants by dot blot (J2 monoclonal antibody, Abcam) and lack of TNF-α production by primary human dendritic cells (Human Immunology Core, University of Pennsylvania). In vitro transcribed mRNAs were used for multiplexing and luciferase immunohistochemistry studies.

### Lipidoid synthesis

Lipidoids were synthesized as previously described (*97*). Specifically, the amines 306 and 200 were reacted with the tail isodecyl acrylate (O_i10_) at a molar ratio of 1:4 to form the lipidoids 306O_i10_ and 200O_i10_, respectively. The amine 514 was synthesized by reacting 2-hexyl-decyl acrylate with sodium cyanide to form a nitrile, which was subsequently reduced to a primary amine with lithium aluminum hydride (*97*). Branched tail O_6,10_ was synthesized by reacting alcohol (2-hexyl-decanol, Sigma-Aldrich) with acryloyl chloride (Alfa Aesar) and trimethylamine (Sigma-Aldrich) in a molar ratio of 1:1.5:2 in reagent grade acetone (Spectrum) in a round bottom flask on ice. Ice was removed after 10 min, and the flask was equilibrated to room temperature for 2 hours. The reaction was quenched with 3 ml of deionized water for 10 min. Product was then rotary evaporated for approximately 1.5 hours, dissolved in ethyl acetate, and placed in a separation funnel. Four washes were done to remove contaminants: (i) NaCl (saturated) and water in 1:1 molar ratio, (ii) 1 M HCl and water in 1:1 molar ratio, (iii) NaHCO_3_ (saturated), and (iv) NaCl (saturated). Product was then retrieved, and 3 to 6 mg of 2,5-di-*tert*-butylhydroquinone (Sigma-Aldrich) was added to prevent polymerization. Magnesium sulfate (Fisher Chemicals) was then added to remove water and then filtered out. Product was rotary evaporated to remove solvent. Tail purification was done using Teledyne ISCO Chromatography with a gradual increase to 40% of dichloromethane:methanol:ammonium hydroxide (60:30:10) in dichloromethane as the model phase and silica as the solid. Structure was confirmed with H-1 and C-13 nuclear magnetic resonance (500 Hz). Amines and tails were combined in glass scintillation vials and stirred at 90°C for 3 days without solvent. The lipidoids were purified using a Teledyne ISCO Chromatography system (Thousand Oaks, CA) to isolate the fully substituted lipidoid product. The structures of the final products are shown in fig. S1.

### LNP formulation

LNPs were formulated as previously described (*20*, *22*). In experiments comparing intravenous and intraperitoneal delivery, the lipidoids 306O_i10_, 200O_i10_, and 514O_6,10_ were diluted with DOPE, cholesterol, and C14-PEG2000 in a molar ratio of 35:16:46.5:2.5 in 90% (v/v) ethanol and 10% (v/v) 10 mM sodium citrate. In experiments comparing amphipathic phospholipids (DOPE, PS, and DOTAP), the lipid molar ratio was adjusted to 35:40:22.5:2.5 lipidoid:helper lipid:cholesterol:C14-PEG2000. mRNA (TriLink) was diluted in 10 mM sodium citrate buffer at pH 4. Equal volumes of lipid and mRNA solution were mixed by rapid pipetting the lipid solution into the mRNA solution and vortexing for 5 s. The final weight ratio of lipidoid:mRNA was 10:1. This yields a nitrogen : phosphate (N/P) ratio of 7.8 for LNPs made with DOPE or PS and an N/P ratio of 8.8 for LNPs containing 40% DOTAP. LNPs were dialyzed against PBS at 4°C overnight in cassettes at a molecular weight cutoff of 3500 g/mol (Thermo Fisher Scientific).

### Animal studies

All animal experiments were conducted using institutionally approved protocols (Institutional Animal Care and Use Committee). C57BL/6 mice (female unless otherwise indicated) were obtained from Charles River Laboratories, Wilmington, MA. STZ mice (males) and NOD and NOD/SCID mice (females) were obtained from the Jackson Laboratory (Bar Harbor, ME). STZ mice exhibited hyperglycemia (blood glucose > 250 mg/dl) upon arrival. In experiments involving clodronate liposomes, clodronate liposomes and control liposomes were purchased from Liposoma (Amsterdam, The Netherlands). Mice received intraperitoneal injections of 200 μl of clodronate or control liposomes 48 hours before LNP treatment. For fluorescence and luminescence studies, dissected organs were imaged using an IVIS (Perkin Elmer). In experiments using mRNA encoding luciferase, mice received an intraperitoneal injection of 130 μl of d-luciferin (30 mg/ml) 15 min before imaging. Blood samples were drawn via submandibular bleed and collected in Microtainer Serum Separator tubes (Becton Dickinson, Franklin Lakes, NJ).

### Immunohistochemistry

Organs for immunohistochemistry were dissected and immediately fixed in 4% paraformaldehyde overnight at 4°C. Fixed samples were dehydrated in 30% sucrose and then embedded and frozen in optimal cutting temperature (OCT) compound (Tissue-Tek, Torrance, CA). Samples were cut into 10-μm sections onto microscope slides and subject to antigen retrieval in sub-boiling citrate buffer at pH 6. Sections were stained with anti-luciferase antibody (NB100-1677, Novus Biologicals, Centennial, CO) or anti-CD45 antibody (70257, Cell Signaling Technology, Danvers, MA) diluted 1:100 in 5% normal goat serum (Thermo Fisher Scientific) overnight at 4°C. Samples were washed in PBS and 0.1% Tween 20 and incubated with horseradish peroxidase–conjugated (luciferase) or Alexa Fluor 488–conjugated (CD45) secondary antibodies diluted 1:100 in 5% normal goat serum for 1 hour at room temperature. Luciferase-stained samples were developed with 3,3′-diaminobenzidine. CD45-stained samples were counterstained with 4′,6-diamidino-2-phenylindole. Slides were visualized using a Keyence BZ-X microscope.

### Flow cytometry analysis of peritoneal immune cells

Peritoneal immune cells were harvested as described previously (*98*). Briefly, mice treated with LNPs containing Cy5-mRNA were euthanized before peritoneal cell harvest. Ice-cold PBS (5 ml; Invitrogen, Carlsbad, CA) supplemented with 5% fetal bovine serum (FBS; Invitrogen, Carlsbad, CA) was injected into the peritoneal cavity, which was gently massaged to dislodge attached cells into the solution. PBS was subsequently recollected into the syringe, ensuring minimal blood contamination, and transferred to tubes kept on ice. Cells were collected by centrifugation at 500*g* and resuspended in PBS and 5% FBS for staining. Cells were stained with the following antibodies in the presence of 1:1000 TruStain FcX antibody (BioLegend): CD19-BV421 (BioLegend, 115537), CD3–fluorescein isothiocyanate (BioLegend, 100203), CD11b-BV650 (BioLegend, 101239), and F4/80-allophycocyanin/Cy7 (BioLegend, 157315), all diluted 1:100. Flow cytometry was performed using a NovoCyte 3000 flow cytometer, and data were analyzed in NovoExpress (Agilent, Santa Clara, CA).

### Cell culture and LNP transfection

Mouse J774A.1 cells [American Type Culture Collection (ATCC), TIB-67, Manassas, VA] were cultured at 37°C and maintained in RPMI 1640 (Gibco, Gaithersburg, MD) supplemented with 10% heat-inactivated FBS (Invitrogen, Carlsbad, CA) and 1% penicillin-streptomycin (Invitrogen, Carlsbad, CA). AlphaTC1 Clone 9 cells (ATCC, CRL-2350, Manassas, VA) were grown in Dulbecco’s modified eagle’s medium (DMEM; Invitrogen, Carlsbad, CA) supplemented with 10% FBS and 1% penicillin-streptomycin. Min6 cells were grown in DMEM/F-12 containing sodium bicarbonate (2.438 g/liter) and sodium pyruvate and supplemented with 10% FBS and 1% penicillin-streptomycin, 285 μM 2-mercaptoethanol, and 2 mM l-glutamine. In cell culture experiments, LNPs formulated with the lipidoid 306O_i10_ and mRNA encoding GFP (TriLink) were delivered to cells at an mRNA concentration of 0.5 μg/ml.

### EV isolation

EV-depleted medium was obtained by centrifuging complete medium (supplemented with 10% FBS) at 100,000*g* for 12 hours and was used for all EV collection experiments. EVs from conditioned media were isolated by SEC (*61*–*63*). Specifically, 1 ml of J774A.1 conditioned medium was centrifuged at 2000*g* for 10 min at 4°C and then at 10,000*g* for 30 min at 4°C. The supernatant was passed through a 0.22-μm pore Millipore filter, and EVs were isolated by mini-SEC using 1.5 cm–by–12 cm mini columns (Bio-Rad, Hercules, CA, USA; Econo-Pac columns) packed with 10 ml of Sepharose 2B (MilliporeSigma, St. Louis, MO) equilibrated with PBS. Conditioned medium (1.0 ml) was loaded onto the column, and five 1-ml fractions corresponding to the void volume peak were collected. Fraction 4 was collected and used for subsequent experiments as the “EV” fraction. Given that SEC is a size-dependent assay, we anticipate that the EVs obtained using this approach contain a heterogeneous mixture, including exomeres, EVs, and microvesicles, in size range of 30 to 200 nm (*99*).

### EV cell uptake studies

Isolated EVs were labeled with DiD membrane-labeling solution (Invitrogen, Carlsbad, CA) using the manufacturer’s instructions. Briefly, EV solution (10 μg/ml) was incubated with 10 μl of DiD solution (5 μmol/liter) for 30 min at 37°C to stain the EV membrane. Excess dye was removed by washing 20× with PBS using 100-kDa Amicon Ultra centrifugal filters (MilliporeSigma, Burlington, MA). AlphaTC or Min6 cells were seeded on coverslips at a density of 5000 cells/mm^2^. DiD-labeled EVs were incubated with cells for 6 hours. To wash off EVs bound to the cell membrane surface, cells were treated with stripping buffer [500 mM NaCl and 0.5% acetic acid in deionized water (pH 3)] for 45 s, followed by three washes with PBS. Cells were fixed with 3.33% paraformaldehyde and labeled with Alexa Fluor 488–Phallodin (5:200 in PBS) and Hoechst 33342 (1:1000 in PBS). Imaging was performed using a Zeiss LSM 880 confocal microscope (Carl Zeiss Microscopy), and the images were processed using ZEN Blue software (Carl Zeiss Microscopy). All experiments were performed in triplicates, and for each individual experiment, five images were taken at random locations. For flow cytometry analysis, AlphaTC or Min6 cells were seeded in 12-well plates at 4 × 10^5^ cells per well and cultured overnight. DiD-labeled EVs were added to the cells for 6 hours. To wash off EVs bound to the cell membrane surface, cells were treated with stripping buffer [500 mM NaCl and 0.5% acetic acid in deionized water (pH 3)] for 45 s, followed by three washes with PBS. Cells were fixed with 3.33% paraformaldehyde and analyzed using NovoCyte 3000 flow cytometer.

### EV-mediated mRNA transfection

J774A.1 cells (4 × 10^5^) were seeded in each well of a 12-well plate and allowed to adhere for 6 hours. Cells were transfected with 50 μl of LNPs [containing GFP mRNA (5 μg/ml)] per well for 6 hours. After transfection, cells were washed with PBS three times (5 min each wash), and 1 ml of EV-depleted medium was added to each well. Cells were incubated at 37°C for 72 hours before collecting the conditioned medium. EVs were isolated from the conditioned medium as described above. AlphaTC cells or Min6 cells were seeded in a 12-well plate at 4 × 10^5^ cells per well and allowed to adhere overnight. Respective treatments [PBS (control)/LNPs/EVs] were added to the wells and incubated for 24 hours. After incubation, cells were washed with PBS, and GFP expression was analyzed using flow cytometry.

### In vivo EV delivery

Primary peritoneal macrophages were used to derive LNP-EVs. Specifically, the abdomens of 6-week-old C57BL/6 female mice were cleaned with 70% alcohol, and a small incision along the midline was made with sterile scissors. Then, abdominal skin was manually retracted to expose the intact peritoneal wall. A 10-ml syringe was filled with ice cold PBS containing 3% exosome-depleted FBS (harvest medium). With the beveled end of a 20-gauge needle facing inward, the needle was inserted through the peritoneal wall along the mouse’s left side (spleen side), and 10 ml of the cold harvest medium was injected into the mouse. Subsequently, the mouse belly was massaged for 2 min to let the harvest media circulate in the intraperitoneal cavity. Using the same syringe and needle, fluid from the peritoneum was aspirated and spun down at 250*g* for 5 min at 4°C. The cell pellet was suspended in RPMI medium supplemented with 10% exosome-depleted FBS, and 6 × 10^6^ cells were seeded in a tissue culture-coated 100-mm petri dish. After letting the cells adhere for 6 to 8 hours, fresh medium supplemented with 300 μl of LNPs (1.5 × 10^12^ particles/ml) containing luciferase mRNA (0.1 mg/ml) was added. LNPs consisted of 35 mole percent (mol %) 306O_i10_, 40 mol % DOTAP, 22.5 % cholesterol, and 2.5 mol % lipid-PEG2000. After 24 hours, cells were washed three times with PBS, and fresh medium was added. After 72 hours, medium (~10 ml) was collected, and EVs were isolated using a combination of centrifugation and SEC (see the “EV isolation” section). Female mice (20 g) were injected intraperitoneally with 100 μl of 1.5 × 10^11^ LNPs [luciferase mRNA (0.5 mg/kg)] or with 100 μl of 1.5 × 10^11^ EVs harvested from primary macrophages. After 3 hours, mice were injected intraperitoneally with d-luciferin potassium salt, euthanized, and dissected for ex vivo luminescence imaging using IVIS.

### Immunogenicity and histology analysis

Female C57BL/6 mice received intraperitoneal injections of LNPs formulated with 306O_i10_, 40% DOTAP, and luciferase mRNA (0.5 mg/kg). For cytokine analysis, blood was drawn via the submandibular vein before injection and 1, 3, 6, and 48 hours after injection, and serum was isolated. For IgM and IgM analysis, serum was collected before injection and 2, 4, 7, 10, and 14 days after injection. ELISAs were performed according to the manufacturer’s instructions using serum dilutions of 1:20 (TNF-α, IL-6, and IL-2) or 1:200,000 (IgG and IgM). For histology, mice were euthanized 2 weeks after injection, and organs were fixed overnight in 4% paraformaldehyde and transferred to 30% sucrose. Samples were embedded in OCT, sectioned, and stained with H&E.

## Notes

View/request a protocol for this paper from *Bio-protocol*.
