# Supplementary material for: Ionizable lipid nanoparticles deliver mRNA to pancreatic β cells via macrophage-mediated gene transfer
Source: Sci Adv. 2023 Jan 27;9(4):eade1444. doi: 10.1126/sciadv.ade1444 (PMC9882987; doi:10.1126/sciadv.ade1444)
Supplement: Supplementary file 1 — Figs. S1 to S8 Supplementary Methods [file sciadv.ade1444_sm.pdf]

Science Advances
AAAS

Supplementary Materials for

Ionizable lipid nanoparticles deliver mRNA to pancreatic β cells via macrophage-mediated gene transfer

Jilian R. Melamed *et al.*

Corresponding author: Kathryn A. Whitehead, kawhite@cmu.edu

Sci. Adv. **9**, eade1444 (2023)
DOI: 10.1126/sciadv.ade1444

This PDF file includes:

Figs. S1 to S8
Supplementary Methods

Ionizable lipid nanoparticles deliver mRNA to pancreatic beta cells via macrophage-mediated transfer

Supplementary Data

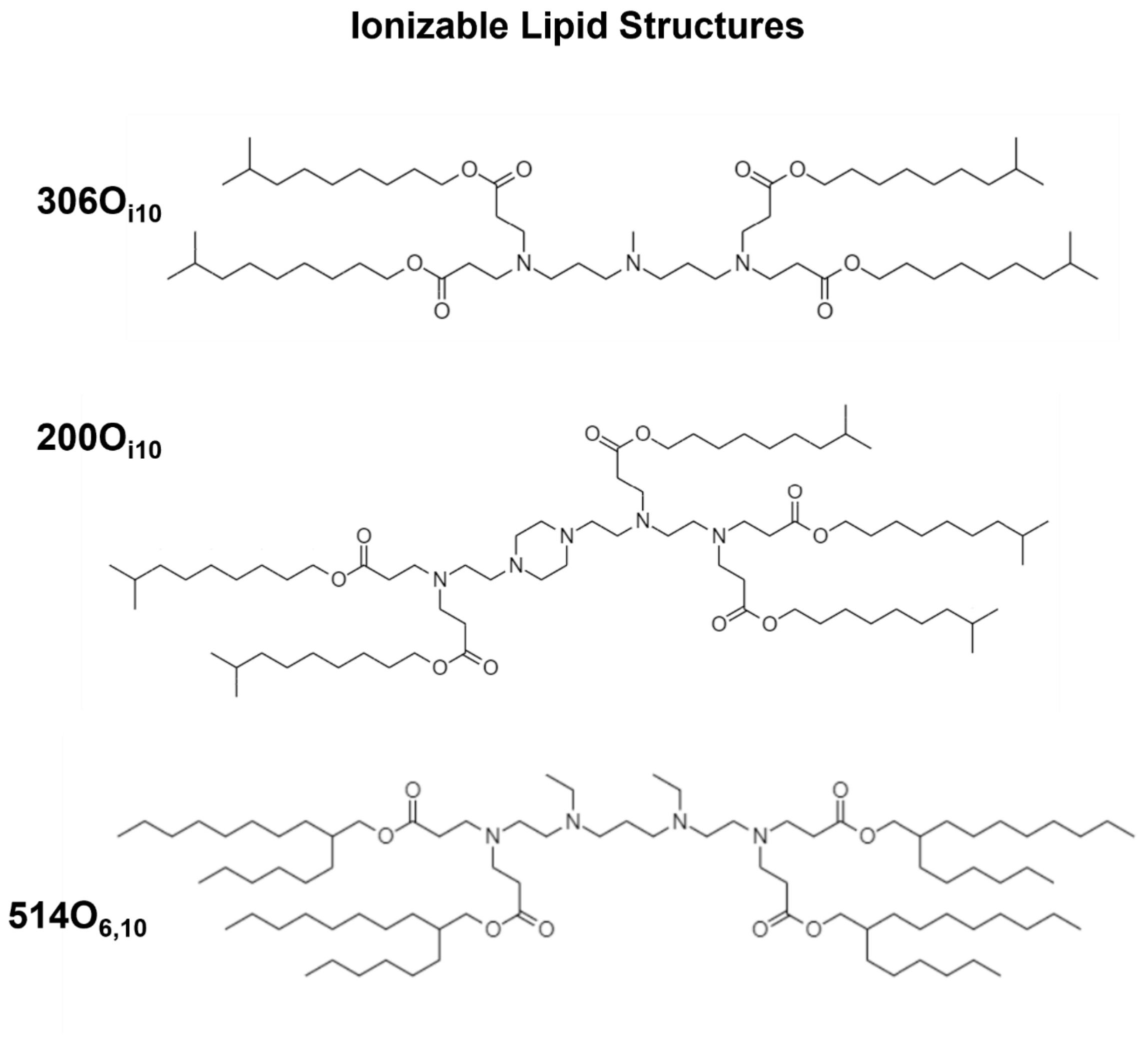

Fig S1: Chemical structures of ionizable lipids.

Phospholipid “Helper Lipid” Structures

DOPE

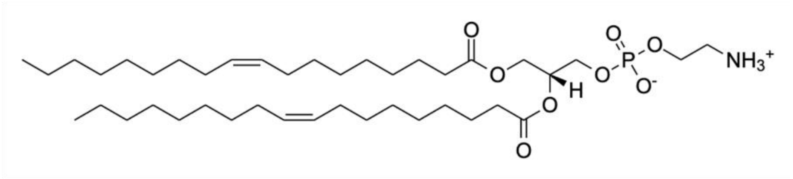

PS

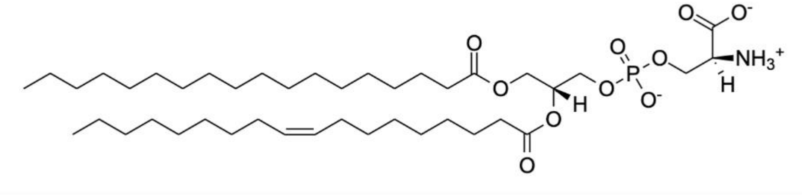

DOTAP

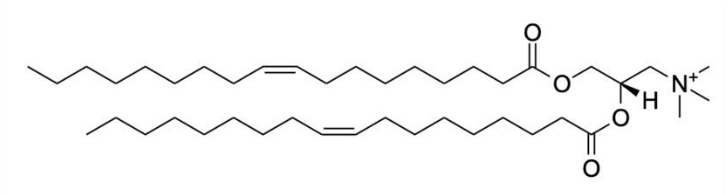

Fig S2: Chemical structures of amphipathic phospholipid helper lipids.

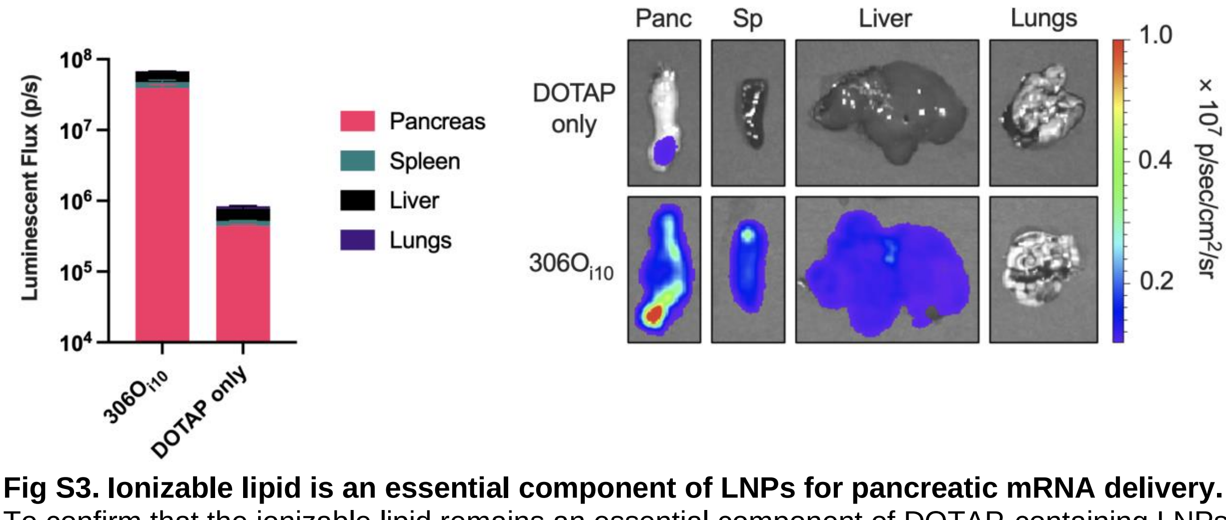

Fig S3. Ionizable lipid is an essential component of LNPs for pancreatic mRNA delivery. To confirm that the ionizable lipid remains an essential component of DOTAP-containing LNPs, we compared DOTAP-containing LNPs with or without $306O_{i10}$. IVIS imaging demonstrates that LNPs containing $306O_{i10}$ and DOTAP outperform DOTAP only LNPs by ~2 orders of magnitude.

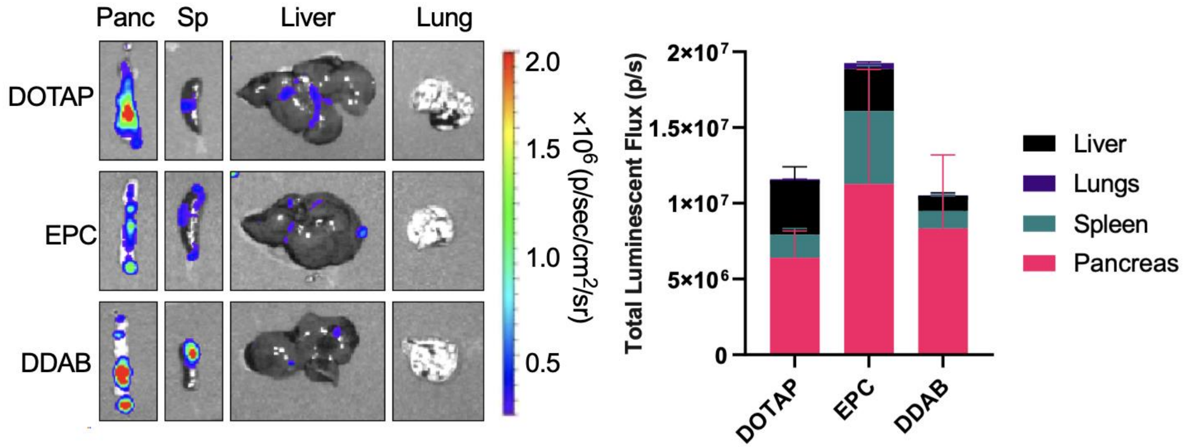

Fig S4. Alternative cationic helper lipids deliver mRNA to the pancreas similarly to DOTAP. 306Oi10 LNPs carrying mLuc were formulated with 40% DOTAP, EPC, or DDAB, then delivered to mice by IP injection. IVIS imaging shows that each helper lipid induces protein expression in the pancreas with similar potency and specificity.

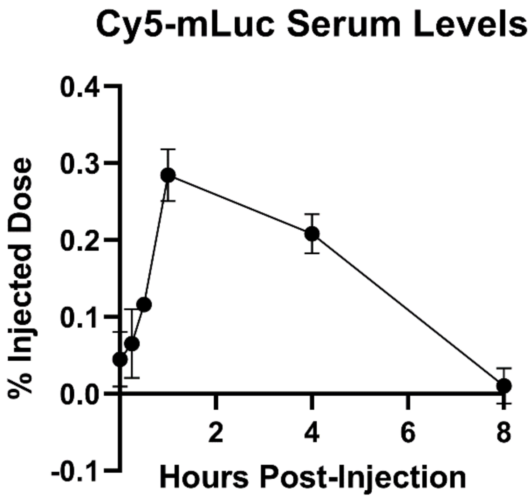

Fig S5: Cy5-mLuc delivered via IP-injected LNPs maximally accumulates in the bloodstream 1 hour post-injection. LNPs formulated with Cy5-mLuc were delivered to mice at a dose of 0.5 mg/kg mRNA. Blood samples were collected in serum collection tubes via the submandibular vein at the indicated timepoints. Serum Cy5 fluorescence was compared to that of a Cy5-mLuc standard curve to determine the quantity of mRNA in the samples.

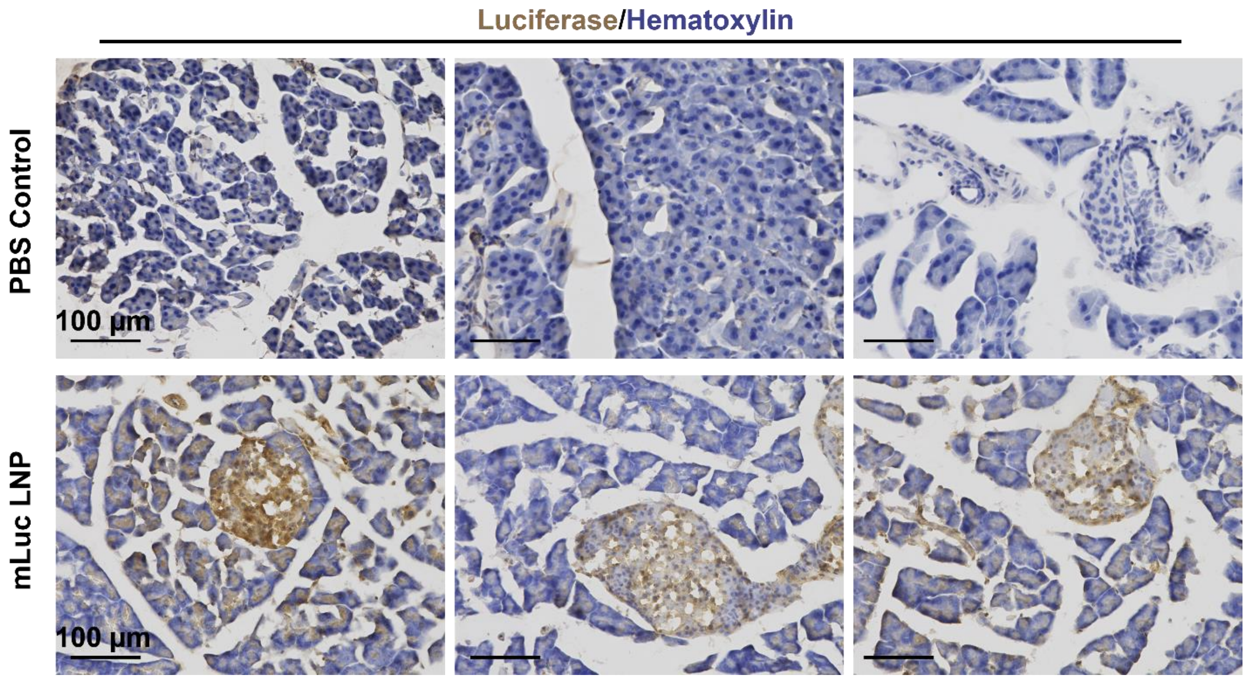

Fig S6: **Luciferase IHC staining (brown) appeared most prominently in islets, with diffuse signal appearing throughout acinar tissue as well.** LNPs were injected at a dose of 0.5 mg/kg mRNA, and organs were dissected and fixed 3 hours post-injection. Images shown were captured at 20X and are representative of 3 independent replicates.

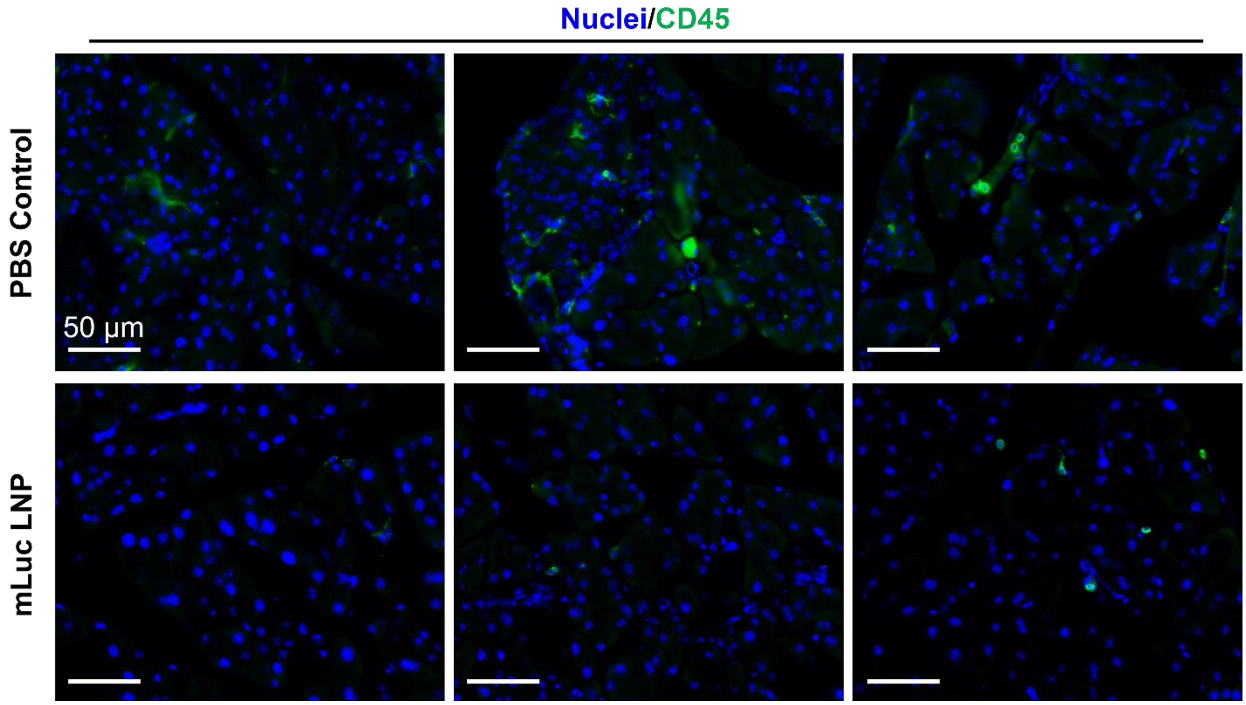

Fig S7: **LNP administration is not associated with an increase in CD45+ cells in the pancreas.** LNPs were injected at a dose of 0.5 mg/kg mRNA, and organs were dissected and fixed 3 hours post-injection. Images shown were captured at 20X and are representative of 3 independent replicates.

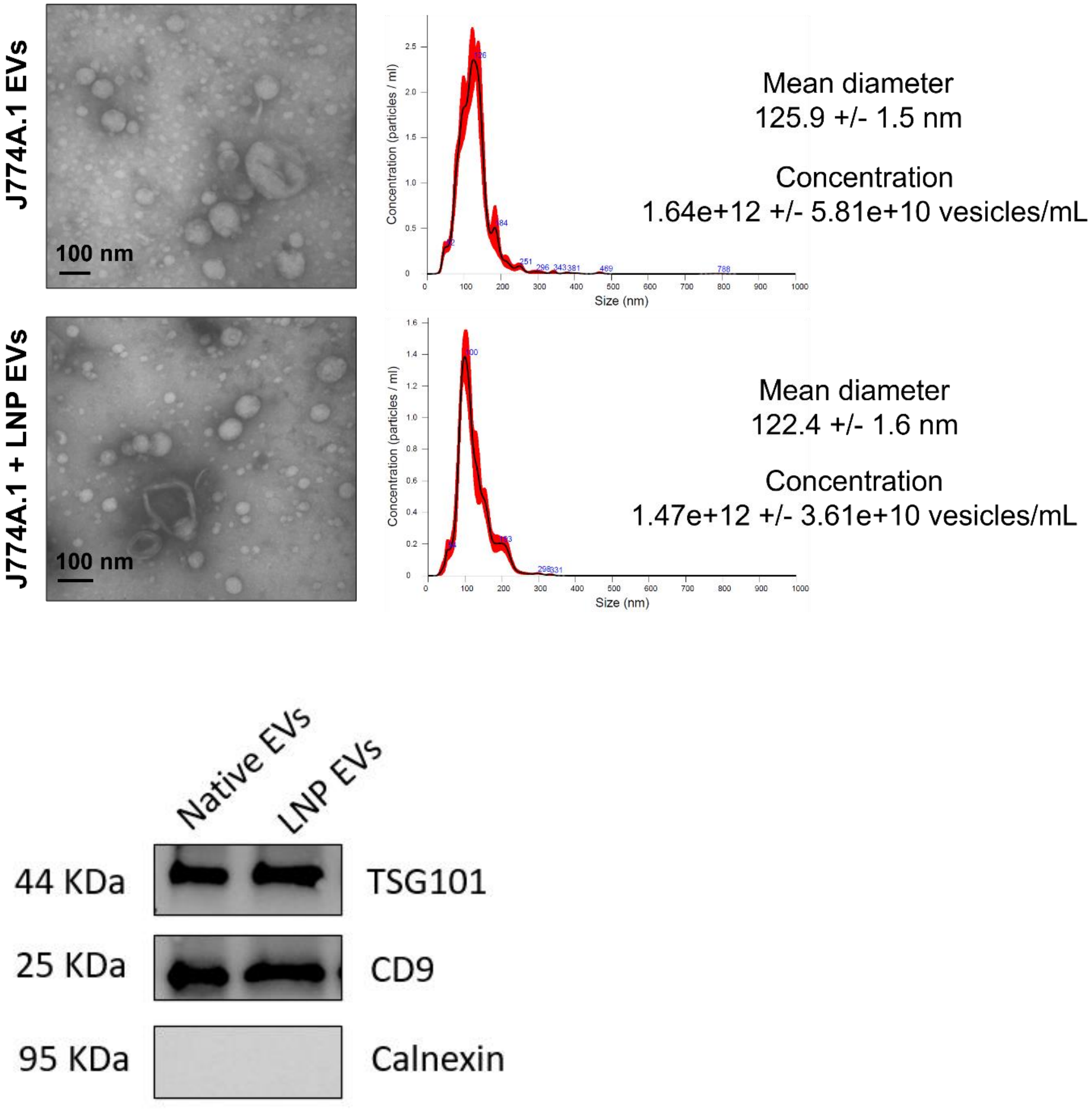

Fig S8: **EV characterization by TEM, NTA, and Western blot.** By TEM and NTA, EVs from untreated and LNP-treated macrophages are ~125 nm in diameter. By Western blotting, EVs are positive for the EV markers TSG101 and CD9 and negative for the endoplasmic reticulum marker calnexin.

Supplementary Methods

Characterization of EVs

Transmission Electron Microscopy (TEM)

EVs were fixed with 4% glutaraldehyde (Electron Microscopy Services, Hatfield, PA, USA) for 20 min at room temperature (RT). A 10 μL droplet of glutaraldehyde-fixed EVs was placed on Formvar-coated 300 mesh copper grid (Electron Microscopy Services, Hatfield, PA). The sample was incubated for 1 min followed by rinsing with distilled water for 1 min to ensure the removal of PBS salts. Excess liquid was blotted off with a Whatman filter. Post rinsing, 50 μL of the uranyl-acetate solution was put on the grid and allowed to remain for 1 min. Excess liquid was removed, and the grids were viewed on a Hitachi H-7100 transmission electron microscope (Hitachi High Technologies) operating at 100 keV. Digital images were collected using a CCD camera system (AMT Advantage 10, Advanced Microscopy Techniques) and inspected using the NIH ImageJ software.

Nanoparticle Tracking analysis (NTA)
EVs were analyzed using the NanoSight LM10 system (NanoSight, Ltd., Amesbury, UK), configured with a 405-nm laser and a high-sensitivity digital camera system (OrcaFlash2.8, Hamamatsu C11440; NanoSight, Ltd.). The camera shutter speed was fixed at 30.01 ms, and camera gain was set to 500. Videos were collected and analyzed using the NTA software (version 2.3), with the minimal expected particle size, minimum track length, and blur setting all set to automatic.

Western Blotting
EVs (15 μg protein after concentration of the collected 1 mL fractions from SEC by VivaSpin 500) were lysed with Laemmli sample buffer (Bio-Rad Laboratories, Hercules, CA, USA), separated on 7–15% SDS/PAGE gels and transferred onto the polyvinylidene fluoride (PVDF) membrane (Millipore, Billerica, MA, USA) for western blot analysis. Membranes were incubated overnight at 4 °C with TSG101 antibody (1:500; catalog # MA1-23296, ThermoFisher Scientific, Waltham, MA) or CD9 antibody (1:500, catalog # ab236630, Abcam, UK) or calnexin antibody (1:500, catalog # ab22595, Abcam, UK). Next, horseradish peroxidase (HRP)-conjugated secondary antibody (1:5000, Pierce, ThermoFisher Scientific, Waltham, MA) was added for 1h at room temperature (RT), and blots were developed with ECL detection reagents (GE Healthcare Biosciences, Marlborough, MA).
